# Imbalance between Endothelial Damage and Repair: A Gateway to Cardiovascular Disease in Systemic Lupus Erythematosus

**DOI:** 10.1155/2014/178721

**Published:** 2014-03-26

**Authors:** Anselm Mak, Nien Yee Kow

**Affiliations:** ^1^Division of Rheumatology, Department of Medicine, University Medicine Cluster, 1E Kent Ridge Road, Level 10, NUHS Tower Block, Singapore 119228; ^2^Department of Medicine, Yong Loo Lin School of Medicine, National University of Singapore, Singapore 119228

## Abstract

Atherosclerosis is accelerated in patients with systemic lupus erythematosus (SLE) and it leads to excessive cardiovascular complications in these patients. Despite the improved awareness of cardiovascular disease and advent of clinical diagnostics, the process of atherogenesis in most patients remains clinically silent until symptoms and signs of cardiovascular complications develop. As evidence has demonstrated that vascular damage is already occurring before clinically overt cardiovascular disease develops in lupus patients, intervention at the preclinical stage of atherogenesis would be plausible. Indeed, endothelial dysfunction, one of the earliest steps of atherogenesis, has been demonstrated to occur in lupus patients even when they are naïve for cardiovascular disease. Currently known “endothelium-toxic” factors including type 1 interferon, proinflammatory cytokines, inflammatory cells, immune complexes, costimulatory molecules, neutrophils extracellular traps, lupus-related autoantibodies, oxidative stress, and dyslipidemia, coupled with the aberrant functions of the endothelial progenitor cells (EPC) which are crucial to vascular repair, likely tip the balance towards endothelial dysfunction and propensity to develop cardiovascular disease in lupus patients. In this review, altered physiology of the endothelium, factors leading to perturbed vascular repair contributed by lupus EPC and the impact of proatherogenic factors on the endothelium which potentially lead to atherosclerosis in lupus patients will be discussed.

## 1. Introduction

### 1.1. Systemic Lupus Erythematosus and Cardiovascular Disease

Systemic lupus erythematosus (SLE) is a systemic autoimmune condition mainly mediated by immune-complex induced inflammation which potentially affects any organ system during the course of the disease [1]. Although the overall survival of lupus patients has been improving in the past 5 decades, excessive mortality is unanimously evident [2]. While disease- and treatment-related complications such as renal disease and infections remain as the leading causes of death in patients with SLE, cardiovascular disease (CVD) is emerging as an increasingly common cause of mortality amongst these patients over the past 30 years [3]. While patients with SLE in general are over 2 times more likely to have CVD as compared with the general populations [4], an epidemiological study revealed that lupus patients older than the age of 35 are >50 times more likely to develop CVD than their age- and sex-matched healthy counterparts [5]. The reasons for the high prevalence of CVD in lupus patients are multifactorial. Besides the fact that patients with SLE carry more unfavourable traditional Framingham risk factors such as hypertension, dyslipidaemia, and diabetes mellitus than their healthy counterparts, nonclassical cardiovascular risk factors, systemic inflammation and proinflammatory adipokines, and treatment-related side effects are operant [6]. While not as extensively studied as in patients with rheumatoid arthritis in larger scale studies [7–10], genetic polymorphisms potentially contributing to cardiovascular disease in patients with SLE have increasingly been identified in a number of lupus cohorts [11–16]. Thus far, genetic polymorphisms associated with premature atherosclerosis and cardiovascular disease in patients with SLE have been convincingly found in the* interferon regulatory factor 8 (IRF8)* [11],* matrix metalloproteinase-2 (MMP-2) functional promoter *[12],* plasminogen activator inhibitor 1 (PAI-1) promoter* [13],* mannose-binding lectin-2 (MBL-2)* [14],* stromelysin promoter* [15], and* C-reactive protein (CRP) *genes [16]. With the ever-increasing knowledge in the pathogenesis of atherogenesis in SLE, more genetic polymorphisms related to CVD in patients with SLE are expected to be identified.

### 1.2. Early Recognition of Atherogenesis in Patients with Systemic Lupus Erythematosus

Currently, therapeutic strategy for CVD is considered “palliative” in that drugs such as antiplatelet agents, anticoagulants, antihypertensives, and statins aim to mitigate cardiovascular risk factors and reduce the probability of future cardiovascular events [17]. In the era of preventive medicine, it is prudent to recognize atherosclerosis early in its progress so that more meticulous monitoring can be instituted and potential primary interventions can be tested, with an ultimate aim to prevent the development of clinically overt cardiovascular complications such as arrhythmias, myocardial ischaemia, and subendocardial and even full-thickness myocardial infarctions. To date, a number of modalities to detect early changes in the process of atherogenesis such as carotid intima-media thickness [18–20], coronary calcium [21–23], speckle-tracking strain echocardiogram [24, 25], and endothelium-dependent vasodilation [26–30] have been reported in patients with SLE and related autoimmune disorders such as scleroderma and systemic vasculitides [18–25]. Amongst these modalities, assessment of the endothelium has received much attention because, at present, it is strongly believed that endothelial dysfunction is one of the earliest steps involved in the process of atherogenesis [31]. Additionally, endothelial dysfunction is theoretically reversible, making it a potentially attractive site of target for preventive intervention against the development of CVD [32]. In this review, how various factors affect the physiology of the endothelium which result in the imbalance between endothelial damage and endothelial repair that lead to CVD, as evident in both murine lupus models and human disease, will be discussed. Information constituting this review was extracted from relevant original papers and review articles on PubMed between 1950 and January 2014 by using the combinations of the keywords “lupus,” “cardiovascular,” and “endothelial.” Bibliography of the relevant articles obtained was thoroughly assessed for relevancy. References which were deemed to be relevant by the authors of this review were further hand-searched, with the useful information extracted for discussion in this paper.

## 2. Normal Physiology of the Endothelium and the Functions of Nitric Oxide—In Brief

The vascular endothelium is a monolayer of cells, which line the luminal surface of blood vessels of all sizes, and confers a physical barrier from potential injuries induced by various vascular toxic components in the blood such as inflammatory mediators, oxidizers, infective agents, and migrating inflammatory cells [33]. Aside from being a physical barrier, the endothelium regulates the vascular tone in response to physiological changes such as intravascular shear stress and high perfusion pressure by producing endothelium-derived relaxing factors (EDRF) which provokes vasodilation and reduces vascular resistance [34]. The EDRF was subsequently identified to be endothelial nitric oxide (NO), which is converted from the substrate L-arginine by the enzymatic action of endothelial NO synthase (eNOS) [35]. NO diffuses into the vascular smooth muscle layer and mediates cyclic GMP-mediated vasodilation. As involved in one of the earliest steps of atherogenesis, the deficiency in the production and bioavailability of NO as a result of endothelial damage lead to impairment of endothelial-dependent vasodilation, which has been proven to be an independent risk factor of cardiovascular events [36, 37].

Besides regulation of vascular tone, NO also contributes to part of the anti-inflammatory and antithrombotic properties on the endothelial level. NO has been demonstrated to reduce interleukin (IL)-1-induced VCAM-1 expression which is paralleled by the reduction of monocyte adhesion to the endothelium, in addition to repressing the production of soluble IL-6 and IL-8 [38, 39]. Recently, an* in vitro* study found that AMP-activated protein kinase, which is central to the regulation of eNOS, reduced TNF*α*-induced monocyte adhesion on human aortic endothelial cells and endothelial MCP-1 expression [40]. As for the antithrombotic effect of NO, it has been demonstrated that the activity of eNOS and the endothelial isoform of NO are critical regulators which suppress platelet activation and aggregation [41].

## 3. Assessment of Endothelial Function: The Current State

### 3.1. Endothelium-Dependent and Endothelium-Independent Flow-Mediated Dilation

To date, there are two established methods to assess the function of the endothelium biophysically, namely, the endothelium-dependent vasodilation, or flow-mediated vasodilation (FMD), and endothelium-independent vasodilation [42]. In brief, for measuring the FMD, subjects are asked to rest in supine position for at least ten minutes before the measurement in the same position. FMD at the brachial artery is measured using a high-resolution ultrasound system, in which the ultrasound probe is steadied by a stereotactic holding device which also permits fine positional adjustment. Reactive hyperaemia is induced by rapid inflation of a pneumatic cuff placed around the proximal forearm to pressure 50 mm Hg above the systolic blood pressure for around 5 minutes, followed by rapid deflation [42]. Change of the vessel diameter at maximum dilatation and percentage of FMD change can hence be detected by the ultrasound probe and calculated by a computer program, with the peak reactive hyperaemic blood flow at 45 to 60 seconds after cuff deflation [42]. All FMD studies are preferably performed after abstention from food and exercise, for 8 to 12 hours, and caffeine and alcohol for 24 and 48 hours, respectively [42]. Another established way to assess endothelial reactivity is to measure endothelium-independent vasodilation of the brachial artery before and after administration of nitroglycerin, which is a direct smooth-muscle relaxant without the need for nitric oxide production and release by the endothelium. After 10 to 15 minutes of rest following completion of endothelium-dependent FMD measurement, 0.4 mg of nitroglycerin, in the form of sublingual spray or tablet, is administered. Peak vasodilation occurs between 3 and 5 minutes after nitroglycerin administration and endothelium-independent FMD can be measured, using the same method as for endothelium-dependent FMD, except that no forearm occlusion is required [43]. According to a recent meta-analysis of 13 studies, FMD but not endothelium-independent vasodilation, is reduced in patients with SLE. However, interpretation of FMD needs to be cautious especially in lupus patients of advanced age and in those who have longstanding SLE because these factors independently affect endothelial function [43].

### 3.2. Endothelial Progenitor Cells

Originated from the haematopoietic stem cells, the endothelial progenitor cells (EPC) are believed to participate in repairing the damaged endothelium and maintaining the integrity of vascular lining [44]. In a number of well-conducted case-control studies, EPC have been shown to be reduced in patients carrying traditional cardiovascular risk factors such as diabetes mellitus and hypertension [45, 46], as well as in those with clinical cerebrovascular and cardiovascular diseases [47, 48]. In a 1-year prospective study of 519 patients with angiography-confirmed coronary artery disease, patients with higher baseline levels of EPC (identified as CD34+CD133+CD309+ cells) were noted to be associated with reductions in risks of death from cardiovascular causes, a first major cardiovascular event, revascularization procedure, and hospitalization by 69%, 26%, 23%, and 38%, respectively, than those with lower baseline EPC levels, after adjusting for age, sex, and vascular risk factors [49].

In rheumatic disorders, EPC have been relatively well studied in scleroderma and SLE, but results are inconsistent [50–54]. The main problem of EPC studies likely stems from the absence of a consensus on the surface markers leading to identification of the true population of EPC, as well as a validated and reliable strategy to identify them. In fact, the European League Against Rheumatism (EULAR) Scleroderma Trials and Research group (EULAR/EUSTAR) has recently proposed a standard method in identifying EPC in patients with scleroderma by the use of fluorospheres and elimination of dead cells and lineage-positive population [51]. Such method resulted in a consistent finding of low levels of circulating EPC in patients with scleroderma [51]. In SLE, while most of the studies demonstrated lower circulating EPC in patients with SLE than their healthy counterparts, results are inconsistent, most likely due to different protocols adopted for identifying EPC [52–54]. Nevertheless, whether the number of circulating EPC can predict cardiovascular events in patients with SLE remains to be answered by prospective studies.

## 4. Altered Physiology of Endothelium in SLE

### 4.1. Factors Associated with Endothelial Damage

Being the hallmark of the pathogenesis of SLE, inflammation has been postulated to be one of the most important triggers of endothelial damage. Type 1 interferon (IFN) appears to play the critical role in mediating endothelial damage in patients with SLE [55], alongside with other endothelial toxic mediators and conditions both dependent and independent of type 1 IFN including proinflammatory cytokines, costimulatory molecules, immune complexes, oxidized lipid species, oxidative stress, autoantibodies, including antiphospholipid antibodies and anti-annexin-V antibodies, and the process of neutrophil extracellular traps (“*Netosis*”).

### 4.2. Type 1 Interferon

Type 1 IFN, which is central to the pathogenesis of SLE and mainly produced by the plasmacytoid dendritic cells (pDC), is increased in the majority of patients with SLE [56, 57]. pDC residing in atherosclerotic plaques produce type I IFN which locally induces adjacent CD4+ T cells to express TNF-related apoptosis-inducing ligand (TRAIL) [58]. While TRAIL was demonstrated to be antiatherosclerotic in the context of chronic inflammation and deficiency of TRAIL was shown to be associated with calcification in atherosclerosis in a mouse model [59, 60], TRAIL potentially leads to plaque rupture and acute coronary event [58]. Type 1 IFN also induces myeloid dendritic cells (mDCs) in atherosclerotic plaques to produce inflammatory cytokines and matrix metalloproteinases which are capable of destabilizing plaques [61]. Platelet aggregation and thrombosis are also induced by type 1 IFN on diseased endothelium in a P-selectin-dependent fashion [62]. Indeed, SLE platelets have been demonstrated to have heightened IFN signatures which are able to activate pDC and subsequent IFN*α* production through the interaction between CD40 and CD40L, potentially perpetuating endothelial toxicity and vascular thrombosis by further activating platelet aggregation as a positive feedback loop [63].

IFN*α* has recently been shown to affect vasculogenesis by interfering the phenotypes and function of EPC [64]. How IFN*α* affects EPC and impairs endothelial repair will be discussed in subsequent sections.

### 4.3. Oxidized Low-Density Lipoproteins and Proinflammatory High-Density Lipoproteins

While epidemiological studies have demonstrated strong associations between high serum levels of circulating oxidized low-density lipoproteins (ox-LDL) and coronary artery disease in the general population [65, 66], a similar association appears to hold true for patients with SLE especially in those with cardiovascular disease and antiphospholipid antibody (APA) syndrome (APS). [67–69]. Mechanistically, ox-LDL induces the secretion of chemokines and proinflammatory cytokines such as monocyte chemotactic protein-1 (MCP-1), IL-8, and IL-6 from the endothelial cells [70]. As a consequence, T cells, monocytes, and dendritic cells are attracted to the subendothelial space of the diseased endothelium where monocytes further differentiate into macrophages that constitute the foam cells under the further stimulation of the ox-LDL [71]. Macrophages, under the influence of IFN*γ* from the T cells, express key proinflammatory cytokines including TNF*α* and IL-1 which in turn aggravate the expression of adhesion molecules on the endothelium including vascular cell adhesion molecule 1 (VCAM-1), intercellular adhesion molecule 1 (ICAM-1), and E-selectin and further attracts monocytes [72]. Additionally, ox-LDL binds to CD14 of macrophages and leads to inhibition of their abilities to phagocytose and induction of CD36 expression [73]. Acting as a scavenger receptor on macrophages, activation of CD36 enhances the uptake of ox-LDL and upregulates the NF-*κ*B expression, perpetuating local inflammatory response [73]. Interestingly, while HDL has long been advocated as the “good cholesterol” in that its level is inversely associated with cardiovascular disease and it functions to reverse LDL and phospholipid oxidation through apolipoprotein (apo A-1) and paraoxonase, respectively, the functions of HDL which can be either anti-inflammatory or inflammatory, is more pathologically relevant in atherogenesis [74]. The proinflammatory form of HDL, which is increased in acute-phase response, has been demonstrated to be able to impair LDL oxidation and the level of proinflammatory HDL was found to be significantly associated with ox-LDL levels in patients with SLE [75].

#### 4.3.1. Atherogenic Adipokines

Amongst various atherogenic adipokines, leptin has been most extensively and systematically studied in patient with SLE, in relation to premature atherosclerosis and cardiovascular disease [76–82]. Leptin is an adipocyte-derived protein which regulates appetite, and energy intake and its expenditure [76]. Plasma leptin was shown to be increased in obese individuals and was correlated with serum C-reactive protein level, endothelial dysfunction, and cardiovascular event [77, 78]. Serum leptin levels have been demonstrated to be higher in patients with SLE as compared with those of healthy individuals [79, 80]. Recently, high serum level of leptin of ≥34 ng/dL has been shown to be associated significantly with carotid plaques with an odds ratio of 7.3 in a study of 210 female patients with SLE and 100 age-matched healthy controls [79]. In the NZB/W F1 mouse model, leptin was shown to enhance survival and proliferation of autoreactive T cells [81] and promote Th17 response through the transcription of Retinoid-Acid Receptor-related Orphan Receptor gamma t (ROR*γ*t) in CD4+ T cells [82]. While further information is required, these findings imply that targeting at leptin would be a potential strategy to combat cardiovascular disease in patients with SLE.

### 4.4. Oxidative Stress

Chronic inflammation of SLE leads to oxidative stress with the production of reactive oxygen species and accumulation of advanced glycation end products which are detrimental to the wellbeing of the endothelium [83]. Recently, it has been demonstrated that under the influence of IFN*α*, cultured lymphocytes undergo intracellular formation of the tubuloreticular structures (TRS) which signifies the presence of oxidative stress on the intracellular level. Additionally, the presence of TRS is significantly and proportionally elevated in higher disease activity of SLE [84]. Thus, it may potentially explain why a number of studies has demonstrated significant associations between clinical disease activity of SLE and various biomarkers of oxidative stress [85–87].

### 4.5. Costimulatory Molecules

#### 4.5.1. CD137-CD137L

CD137 (4-1BB) belongs to the TNF superfamily which is mainly expressed on activated T cells and natural killer T cells. Its ligand, CD137L, is constitutively expressed on antigen presenting cells (APC) including B cells and dendritic cells (DC) [88, 89]. CD137 is a potent costimulatory receptor molecule and its cognate interaction with CD137L induces proliferation of activated T cells, and profound immunoglobulin production in B cells as well as maturation of DC on which CD137L is expressed [89]. Interestingly, agonizing CD137 with anti-CD137 monoclonal antibodies alleviates glomerulonephritis and improves mortality in MRL/lpr mice alongside with reduction of anti-dsDNA antibody, CD4+ T cells, and germinal centre formation [90]. In NZW/B mouse model, agonizing CD137 leads to the alleviation of lupus-like manifestations by increasing splenic CD4+CD25+ T regulatory cells [91].

Endothelial cells have also been shown to express CD137 upon activation and stimulation by proinflammatory stimuli such as TNF*α* [92]. The interaction between CD137 on the endothelium and CD137L expressed on monocytes enhances the former to express adhesion molecules such as ICAM-1 and VCAM-1, and the latter to migrate to vascular wall in an E-selectin and ICAM-1-dependent fashion [92–94]. Thus, CD137 activation promotes atherosclerosis early on the endothelium level. Although agonistic anti-CD137 antibody demonstrates alleviation of lupus in animal models [90, 91], its potential to cause atherosclerosis may be a relevant concern if this monoclonal antibody is to be evaluated in clinical trials for the treatment of SLE.

#### 4.5.2. CD40-CD40L

Similar to CD137L, CD40L belongs to the TNF superfamily and is expressed on T cells [95, 96]. The CD40L gene is a SLE susceptible gene which is overexpressed in female lupus patients, partly due to the consequence of demethylation of the regulatory sequence on the inactivated X chromosome of T cells [95, 96]. CD40L and CD40 interaction between T cells and CD40-expressing endothelial cells triggers endothelial expression of adhesion molecules such as VCAM-1 [97]. While antagonizing CD40L with anti-CD40L in LDL-receptor deficient mice has been shown to cause substantial reductions of atherosclerotic lesions and their lipid content, and the amount of intralesional macrophages and T cells, as well as VCAM-1 expression on the endothelium [98], a clinical trial testing anti-CD40L in patients with SLE was unfortunately terminated prematurely due to excessive occurrence of cardiovascular events [99]. Thus, it seems unlikely that anti-CD40L will be able to protect the cardiovascular system in human SLE even though promising results in alleviating lupus nephritis was evident [99].

### 4.6. Proinflammatory Cytokines

Key proinflammatory cytokines which have been advocated to play a role in endothelial damage include IL-17, IFN-gamma (IFN*γ*), and TNF*α*. Expansion of the Th17 population and elevation of serum IL-17 levels have been clearly demonstrated in patients with SLE [100]. In nonlupus models, IL-17 has been implicated in the development of atherosclerotic plaques. Indeed, depleting IL-17R by knocking out the IL-17R gene of LDL receptor-deficient atherosclerosis-prone mice reduced the size of aortic atherosclerotic plaques in these mice fed with Western-type diet [101]. In humans, T cells which produce both IL-17 and IFN*γ* were demonstrated to reside in the specimens of atherosclerotic plaque from patients with coronary heart disease [102]. Furthermore, in patients with acute coronary syndrome, higher number of circulating Th17 cells and levels of IL-17 as well as its related cytokines such as IL-6 and IL-23 levels were demonstrated as compared with those with stable angina and healthy individuals [102]. Nevertheless, data addressing whether IL-17 is directly related to clinical cardiovascular events are sparse.

As far as TNF*α* is concerned, our team has recently demonstrated that TNF*α* is elevated in patients with SLE with the use of the multiplex immunoassay platform, as compared with age- and sex-matched healthy individuals [103]. TNF*α* elevation was shown to be associated with higher coronary calcium scores in patients with SLE [104]. TNF*α* induces adhesion molecules expression on, and enhances the recruitment of T cells and monocytes to the endothelial cells [105]. As for IFN*γ* which has been discussed above, it is expressed by activated T cells and other immunocytes and it induces the macrophages to express TNF*α* and IL-1 which in turn aggravate the expression of VCAM-1, ICAM-1, and E-selectin and further attracts monocytes towards the diseased endothelium [72]. After all, IFN*γ per se* promotes oxidative stress and resultant endothelial damage [83].

### 4.7. Autoantibodies against ox-LDL, Phospholipids, and Annexin-V in Systemic Lupus Erythematosus

By intuition, antibodies against ox-LDL may alleviate the toxic effect of ox-LDL on the endothelium. Indeed, animal studies revealed that infusion of anti-LDL protected against atherosclerosis in hypercholesterolemic mice [106] and immunization of modified LDL, which triggered high titre of anti-ox-LDL antibodies, reduced atherosclerotic lesions in LDL-receptor deficit rabbits [107]. However, the results do not appear to be translated to human disease. While the cross-reactivity between antibodies against cardiolipin (a phospholipid species) and ox-LDL might imply an increased chance of the development of CVD in patients with SLE, the association between anti-ox-LDL antibodies and CVD remains inconsistent in these patients [67, 108, 109]. On the other hand, the association between antiphospholipid antibodies and CVD is undoubtedly clear. Formation of immune complexes involving *β*2-glycoprotein 1 has been shown to be detrimental to the vascular wall in part due to the stimulation of adhesion molecule expressions on the endothelium [110].

Annexin-V is a naturally occurring and potent phospholipid-binding anticoagulant protein which protects the endothelium from damage by inhibiting the procoagulant effects of tissue factors and binding to negatively charged phospholipids [111, 112]. In patients with SLE, besides the higher levels of anti-annexin-V antibodies, serum anti-annexin-V levels were shown to be predictive of poorer endothelial function gauged by endothelium-dependent vasodilation [111–113]. Mechanistically, it is evident that the binding of the atheroprotective annexin-V to phospholipid bilayer of the endothelium is interfered by the anti-*β*2-glycoprotein 1 antibodies [114].

### 4.8. Immune Complexes

Autoantibodies, which are characteristically abundant in SLE, form immune complexes (IC) with their respective autoantigens. Complements are subsequently fixed onto the IC in an attempt to be opsonised for removal by phagocytes. In fact, complement-associated immune complexes induce endothelial expression of adhesion molecules which enhance migration of T cells and monocytes towards the subendothelial space that initiate endothelial damage [115]. Interestingly, not all IC are detrimental to the wellbeing of the endothelium. C1q complexes are indeed atheroprotective in that they are able to trigger clearance of oxLDL by macrophages [116]. Thus, qualitative and quantitative deficiency of C1q found in patients with SLE may be implicated as a risk factor for CVD.

### 4.9. Neutrophil Extracellular Traps

A recent breakthrough in the research of antimicrobial mechanism by neutrophils is the discovery of the formation of neutrophil extracellular traps (NETs) [117]. NETs essentially comprise intracellular antimicrobial proteins such as LL37 and human neutrophils peptide and DNA which are microbicidal. In patients with SLE, the presence of antibodies against ribonucleoproteins and those against LL37 prime and increase the propensity of NET formation when compared to healthy individuals [118]. In addition, NETs confer strong cytotoxic signals which lead to endothelial damage and endothelial apoptosis [119]. Furthermore, NETs have been shown to activate platelets which induce thrombosis at the site of vascular injury and induce IFN*α* production by pDC which are activated by NETs-stimulated lupus neutrophils [120]. As a result, the tendency of NET formation in patients with SLE results in direct endothelial apoptosis and damage which are further potentiated by its effects on platelet and pDC activation, enhancing vascular thrombosis and perpetuation of the vicious cycle of endothelial dysfunction.

### 4.10. Factors Associated with Perturbed Vascular Repair

#### 4.10.1. Endothelial Progenitor Cells

The serum levels of IFN*α*, and transcription of genes which enhance the expression of those encoding IFN*α* (“IFN signatures”), are upregulated in patients with SLE. IFN*α* plays a central role in the pathogenesis of SLE and, at the same time, it is “toxic” to the endothelium [121]. For example, EPC were demonstrated to undergo striking apoptosis after treating with IFN*α*, accompanied by a reduced capability to differentiate into mature endothelial cells, which were reversible by neutralizing IFN*α* [122]. It has been postulated that type 1 IFN leads to perturbed vascular repair by repressing the expression of angiogenic factors such as VEGF and IL-1*β* on the endothelium, coupled with enhanced expression of IL-18 [123]. Very recently, angiogenic T cells (Tang), a novel subset of T cells which are functionally similar to the EPC in terms of the ability of endothelial repair, have been described in patients with rheumatoid arthritis [124]. Tang express CD3, CD31, and CD184 and their number in the peripheral blood was found to be correlated with that of the EPC in patients with rheumatoid arthritis [124]. Interestingly, the number of circulating Tang was associated positively with the positivity of antinuclear antibody and serum IFN*α* level and negatively with the occurrence of cardiovascular events in 103 patients with rheumatoid arthritis [124]. Since positivity of antinuclear antigen, high IFN*α* level and propensity to develop cardiovascular disease are evident in patients with SLE, phenotypic and functional studies of Tang in lupus patients in relation to cardiovascular disease would potentially yield exciting information of translational potential.

In animal lupus models, NZW/B mice were shown to have impaired endothelium-dependent vasorelaxation, reduction in the quantity, and increase in apoptosis of bone marrow and splenic EPC as compared with BALB/c controls. In addition, EPC from NZW/B failed to differentiate into mature endothelial cells as what C57BL/6 mice did. Type 1 IFN signatures were increased in EPC of NZW/B mice and IFN*α* was shown to induce apoptosis of EPC* in vivo* [125]. Interestingly, B6/lpr mice did not demonstrate quantitative, phenotypic, and functional abnormalities of EPC. While it gives researchers the information that B6/lpr mice might not be an ideal murine model to study endothelial physiology in lupus, lupus activity and renal dysfunction, which are more prominent in the B6/lpr mice, are not the sole contributors to endothelial dysfunction. Locally produced IFN*α* can induce uptake of ox-LDL into macrophages.

Besides the pDC which are the major producer of IFN*α* in patients with SLE, low-density granulocytes (LDGs), which are elevated in patients with SLE, have been demonstrated to produce type 1 interferon sufficiently enough to impair vascular repair [126]. In fact, depletion of LDGs instead of pDC in patients with SLE was shown to restore the capability of EPC to differentiate into normal endothelial monolayers [126].

## 5. Conclusion

Recognition of atherogenesis early in the pathogenesis taking place in the endothelium, exploration of the value of FMD and circulating EPC, and research for potential intervention to maintain the wellbeing of the endothelium before clinical cardiovascular disease develops are potentially useful and highly relevant in reducing cardiovascular mortality and morbidity. Type 1 IFN, which is important to the pathogenesis of SLE, appears to be crucial in initiating and perpetuating endothelial damage and impairing vascular repair through its inhibitory action in EPC. Supported by a prevalent study that high IFN signature is associated with endothelial dysfunction, high coronary calcium score and carotid IMT after controlling for traditional cardiovascular risk factors [127], suppression of type 1 IFN in selected patients with heightened IFN signature might therefore be an attractive avenue in preventing cardiovascular disease in patients with SLE. However, stronger evidence from prospective studies which advocates the association between heavy IFN signatures and development of cardiovascular disease amongst lupus patients is undoubtedly required. In addition, much more work needs to be done to further obtain and validate available knowledge in order to translate it into potentially beneficial therapeutic and preventive interventions against cardiovascular disease in patients with SLE.  Table 1 summarizes the factors and their potential mechanisms which contribute to endothelial damage and impaired endothelial repair in SLE.

## Figures and Tables

**Table 1:** A summary of the factors and their mechanisms which contribute to endothelial damage and impaired repair of the endothelium.

Endothelial damage	Description (ref)
Type 1 interferon	Chiefly produced by pDC, IFN*α* is increased in SLE [46, 47] and it stimulates CD4+ cells residing in atherosclerotic plaque to express TRAIL which in turn enhances plaque rupture [48]. IFN*α* induces mDC residing in atherosclerotic plaques to express proinflammatory cytokines and MMPs which destabilize plaques and promote plaque rupture [49]. IFN*α* stimulates platelet aggregation and vascular thrombosis in a P-selectin dependent fashion [50].

Type 2 interferon	Type 2 IFN is produced by a wide range of immunocytes including mDC, activated lymphocytes, and monocytes. It induces monocytes to upregulate IL-1 and TNF*α* which induce the expression of adhesion molecules such as VCAM-1, E-selectin, and ICAM-1 on the endothelium [59].

Proinflammatory cytokines	Major proinflammatory cytokines, including TNF*α*, IL-1, IL-17, and IFN*γ* which are elevated in SLE, stimulate endothelial expression of adhesion molecules and lead to recruitment of atherogenesis-enhancing monocytes and T cells to the subendothelial space [59, 85]. A clinical study revealed that higher serum TNF*α* levels were associated with higher coronary calcium score [84], a radiological predictor of coronary artery event.

Immune complexes	Complement-fixed immune complexes upregulate the expression of adhesion molecules on the endothelium [96] However, C1q-containing immune complexes are atheroprotective as they promote clearance of ox-LDL by macrophages [97].

Costimulatory molecules	Endothelium expresses CD137 upon activation by proinflammatory signals such as TNF*α* [72]. Ligation of endothelial CD137 with CD137L expressed on monocytes induces the former to express adhesion molecules and facilitate monocyte migration to the subendothelial space [72–74]. CD40 is expressed on the endothelium, and its interaction with CD40L expressed on T cells induces expression of VCAM-1 which enhances atherosclerosis [77]. A clinical study testing anti-CD40L was however terminated due to the unexpected excessive occurrence of cardiovascular events [79].

Oxidized lipids	Circulating ox-LDL induces endothelial secretion of MCP-1, IL-8, and IL6 which attract DC, T cells, and monocytes. Monocytes are induced to form foam cells under the further influence of ox-LDL and proinflammatory cytokines [58].

Oxidative stress	Oxidative stress increases with higher disease activity of SLE [83]. Reactive oxygen species formed during oxidative stress lead to accumulation of glycation end products which are toxic to the endothelium [63].

Autoantibodies	Annexin-V is a naturally occurring phospholipid-binding anticoagulant protein. Lupus patients demonstrate elevation of the anti-annexin-V antibody, which is related to inferior endothelial function [92–94]. Indeed, antiphospholipid antibodies, in particular the anti-*β*2-glycoprotein-1 antibodies, interferes the binding between the atheroprotective annexin-V to the phospholipid bilayer of the endothelium [95].

NETs	Antibodies against ribonucleoproteins and LL37 promote NET formation, which induces IFN*α* production by pDC as result of NETs-stimulated lupus neutrophils [99–101], NETs formation leads to activation of vascular thrombosis and endothelial apoptosis [99–101].

Perturbation of vascular repair	Description (ref).

Endothelial progenitor cells	IFN*α* induces EPC apoptosis and the ability of EPC to differentiate to mature endothelium [102, 103]. Vascular repair is impaired by the ability of IFN*α* to repress VEGF and IL-1 and upregulate IL-18 on the endothelium [104]. LDG is another source of IFN*α* apart from pDC which is elevated in patients with SLE. LDG impairs endothelial cell repair, and depletion of LDG restores the ability of EPC to differentiate into mature EPC and repair the endothelial monolayer [106].

ref: references; pDC: plasmacytoid dendritic cells; IFN*α*: interferon-alpha; SLE: systemic lupus erythematosus; TRAIL: TNF-related apoptosis-inducing ligand; mDC: myeloid dendritic cells; MMP: matrix metalloproteinases; IFN: interferon; IL: interleukin; TNF*α*: tumour necrosis factor-alpha; VCAM-1: vascular cell adhesion molecule 1; ICAM-1: intercellular adhesion molecule 1; IFN*γ*: interferon-gamma; ox-LDL: oxidized low-density lipoproteins; MCP-1: monocyte chemotactic protein-1; DC: dendritic cells; NETs: neutrophil extracellular traps; LDG: low-density granulocytes.
